# Optimizing Portal Venous Phase Acquisition Quality of Contrast-Enhanced CT in Oncology

**DOI:** 10.3390/diagnostics16203372

**Published:** 2026-10-16

**Authors:** Amir Zemmouri, Marcus Talke, Marie Porte, Tania Moussa, Christophe Massard, Gabriel Garcia, Remy Barbe, Nathalie Lassau, Arnaud Dupont, Amandine Crombé, Douraied Ben Salem, Capucine Baldini, Tarek Aissi, Mabel Nuernberg, Corinne Balleyguier, Laurent Dercle, Samy Ammari

**Affiliations:** 1 Département d’Imagerie Médicale, Gustave Roussy Cancer Campus, Université Paris-Saclay, 94800 Villejuif, France; 2 Department of Internal Medicine, New York-Presbyterian/ Columbia University Irving Medical Center, New York, NY 10032, USA; mpt2140@cumc.columbia.edu; 3 Département d’Innovation Thérapeutique et d’Essais Précoces (DITEP), Gustave Roussy Cancer Campus, Université Paris-Saclay, 94805 Villejuif, France; 4 Biomaps, UMR1281, INSERM, Centre National de la Recherche Scientifique (CNRS), Commissariat à l’Energie Atomique (CEA), Université Paris Saclay, 94800 Villejuif, France; 5 Department of Radiology, Pellegrin University Hospital, 33000 Bordeaux, France; 6 Bordeaux Institute of Oncology, BRIC U1312, INSERM, Team ‘SARCOTARGET’, 33000 Bordeaux, France; 7 Department of Neuroradiology, Brest University Hospital, LaTIM (INSERM UMR 1101), Faculty of Medicine and Health Sciences, Université de Bretagne Occidentale, 29609 Brest Cedex, France; 8 Division of International Patients Care, Gustave Roussy Cancer Campus, 94800 Villejuif, France; 9 Department of Radiology, New York-Presbyterian/Columbia University Irving Medical Center, New York, NY 10032, USA; 10 Department of Radiology, Memorial Sloan Kettering Cancer Center, New York, NY 10065, USA

**Keywords:** computed tomography, phase I, clinical trial, neoplasms, contrast media, RECIST 1.1, quality control, legislation, artificial intelligence, photon-counting CT

## Abstract

**Background**: Portal venous phase (PVP) contrast-enhanced CT is a cornerstone for response assessment in early-phase oncology trials, yet contrast-enhancement phase timing remains insufficiently monitored across sites. We assessed PVP timing using a validated artificial intelligence (AI) quality control tool (CECT-QC). **Methods**: In this retrospective multicenter study, 402 CT examinations from 402 patients enrolled in phase I oncology trials across three European institutions were analyzed using CECT-QC, which quantifies aortic and portal venous enhancement to classify contrast-enhancement phase. The primary analysis included 335 standard CT examinations. Optimal PVP classification was compared (i) across institutions, (ii) before vs. after French regulatory changes impacting contrast delivery, and (iii) across scanner technologies (photon-counting CT [PCCT] vs. standard CT). **Results**: Among the 335 standard CT examinations included in the primary analysis, 53.1% (178/335) were classified by CECT-QC as optimal PVP. Optimal PVP rates were 39.0% (32/82) at Institution A, 56.9% (87/153) at Institution B, and 59.0% (59/100) at Institution C, with a significant adjusted institutional association (*p* = 0.039). No significant temporal association was observed before versus after the legislative change (*p* = 0.716). At Institution A, standard CT was associated with higher odds of optimal PVP classification than PCCT (OR 2.24, 95% CI 1.07–4.68; *p* = 0.031), while PCCT showed higher aortic and portal venous attenuation (both *p* < 0.001). **Conclusions**: Approximately half of standard CT examinations in phase I oncology trials were classified as optimal PVP. Classification varied significantly across institutions and by scanner technology, whereas no significant temporal association was observed before versus after the legislative change. These findings support continued quality-control monitoring, protocol harmonization, and adaptation of classification tools to emerging CT platforms.

## 1. Introduction

Reliable response assessment in oncology using CT imaging starts before the first caliper measurement: with a correctly timed and sufficiently enhanced contrast phase. In oncology CT, suboptimal enhancement degrades lesion conspicuity and boundary definition, introducing avoidable variability in RECIST 1.1 measurements and in density-based or composite endpoints such as CHOI [1,2,3,4,5,6]. Even modest deviations in timing can translate into meaningful differences in tumor delineation and attenuation, ultimately influencing downstream clinical decisions and trial interpretation [3,7,8].

This issue is particularly consequential in phase I oncology trials. Contrast-enhanced CT is often the earliest window into therapeutic activity, lack of benefit, or unexpected toxicity [1,9,10,11,12]. Consequently, early-phase units operate under stringent imaging requirements set by sponsors, contract research organizations (CROs), and regulatory agencies. Yet in Europe, these specialized centers remain relatively few, making consistent delivery of standardized, high-quality imaging a critical asset.

In practice, however, patients enrolled in early-phase trials are imaged across heterogeneous settings (academic hospitals, affiliated sites, and private practices), where contrast administration protocols and quality assurance resources vary [13,14,15,16,17,18]. This heterogeneity raises concerns about the robustness of CT-based evaluations and complicates multicenter comparability.

Against this backdrop, recent cost-containment policies may unintentionally amplify variability. In France, the 2024 Social Security Financing Act (LFSS 2024, Article 59) and its implementing decree shifted contrast media procurement toward multi-patient utilization models, while limiting reimbursement pathways for pharmacy-dispensed contrast [19]. While designed to reduce waste and spending [20], these changes may increase the risk of underdosing, protocol deviations, and inconsistent injection timing—particularly in settings without dedicated quality control.

Using France as a case study, we leveraged a validated artificial intelligence (AI) quality-control tool (CECT-QC) that quantifies enhancement in the aorta and portal vein to objectively benchmark portal venous phase (PVP) timing and phase classification [3,21]. We aimed to quantify the rate of optimal PVP enhancement in phase I oncology trial CTs across three European sites, and to compare PVP phase classification (i) between institutions, (ii) before versus after LFSS 2024 implementation, and (iii) between photon-counting CT and standard CT—highlighting the role of scalable quality control in protecting imaging endpoints in early-phase research.

## 2. Methods

### 2.1. Study Population

We retrospectively analyzed 402 CT examinations from 402 unique patients enrolled in phase I oncology trials acquired between June 2022 and February 2025. Only one CT examination per patient was included in the final study cohort. When more than one examination met the eligibility criteria for the same patient, one examination was randomly selected without applying any additional clinical or imaging-based selection criterion. Scans were obtained at three anonymized institutions: a French non-profit tertiary cancer center (Institution A, *n* = 149), a private for-profit French radiology department (Institution B, *n* = 153), and a non-French European tertiary hospital not subject to the French legislative reform (Institution C, *n* = 100). Additional demographic characteristics, scanner types, and primary tumor sites across the three institutions are summarized in Table A1 (Appendix A). Institutions A and B were subject to the new French legislation. For inclusion, eligible patients had confirmed malignancy, available PVP CT scans, and complete clinical and imaging metadata. Scans from subjects with missing weight or contrast dose and concentration data were excluded, as these parameters are essential for normalization and interpretation of the study data. This retrospective study was approved as research not involving human participants by the Scientific Commission for Therapeutic Trials at Gustave Roussy (registration no. 2025-646). Data used for the analysis were anonymized, and informed consent was waived.

### 2.2. Data Collection

For each patient, the following variables were extracted: age, sex, BMI, type and concentration of ICA, administered volume, and scanner model. All subjects were administered high-concentration ICAs (≥350 mg/mL), with weight-adjusted dosing standardized at 0.5 gI/kg. Institution A was equipped with both conventional CT scanners and a photon-counting CT scanner (NAEOTOM Alpha, Siemens Healthineers, Forchheim, Germany), whereas Institutions B and C used conventional CT scanners from various manufacturers and generations. For conventional CT examinations, acquisition protocols used tube voltages in a range comparable to that represented in the original CECT-QC development and validation data. However, individual tube-voltage values were not collected in the present retrospective dataset and therefore could not be summarized by institution or included in adjusted or sensitivity analyses. All PCCT examinations were acquired using a standardized oncologic thoraco-abdomino-pelvic protocol, with virtual monoenergetic image (VMI) reconstructions at 70 keV used consistently for all PCCT examinations. Radiological examinations were categorized by site and date of acquisition. For Institutions A and B, scans were further stratified according to the date of implementation of the French legislative reform (before 1 March 2024 vs. on or after 1 March 2024).

To promote collaborative data validation and reproducibility, a secure environment was used. This included an anonymized spreadsheet for clinical and imaging variables, and real-time cross-institutional communication.

### 2.3. Imaging Analysis

**Figure 1:**
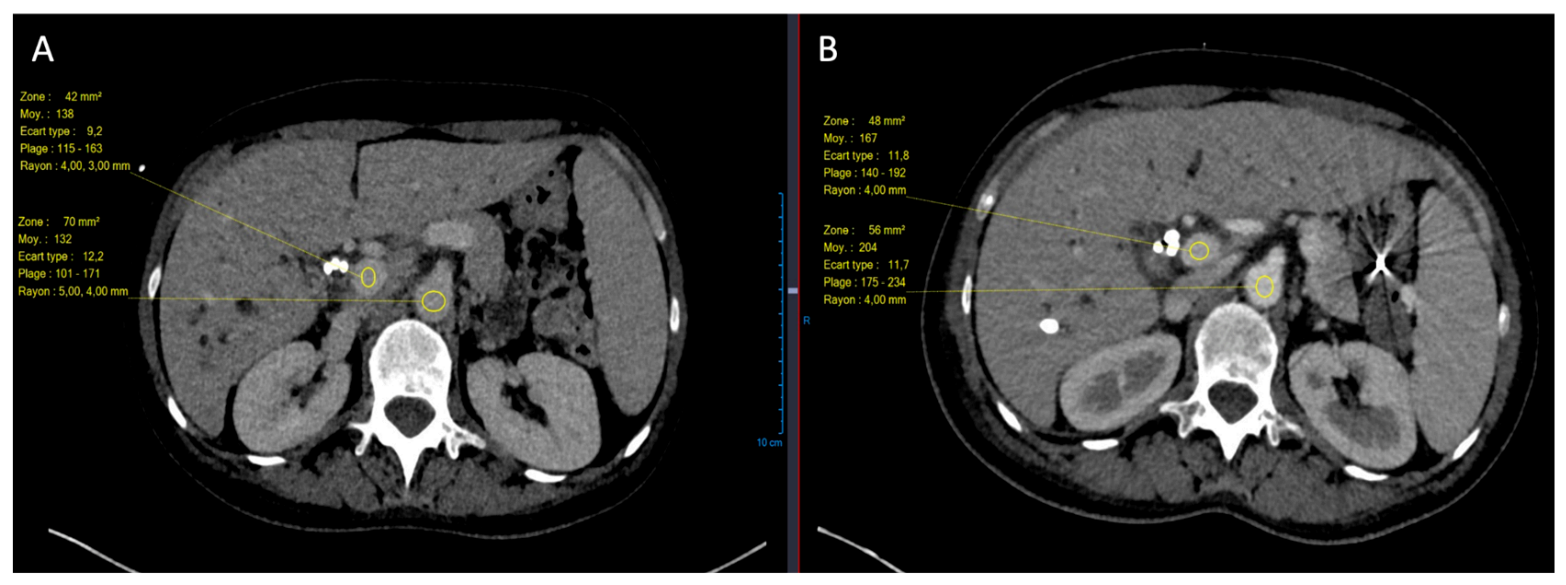
Illustration of automatic segmentation of the aorta and portal vein for contrast enhancement measurement. Contrast-enhanced CT scans from two patients (**A**,**B**) showing the abdominal aorta and portal vein ROIs segmented to measure vascular enhancement in Hounsfield Units, used as input for the AI algorithm.

**Figure 2:**
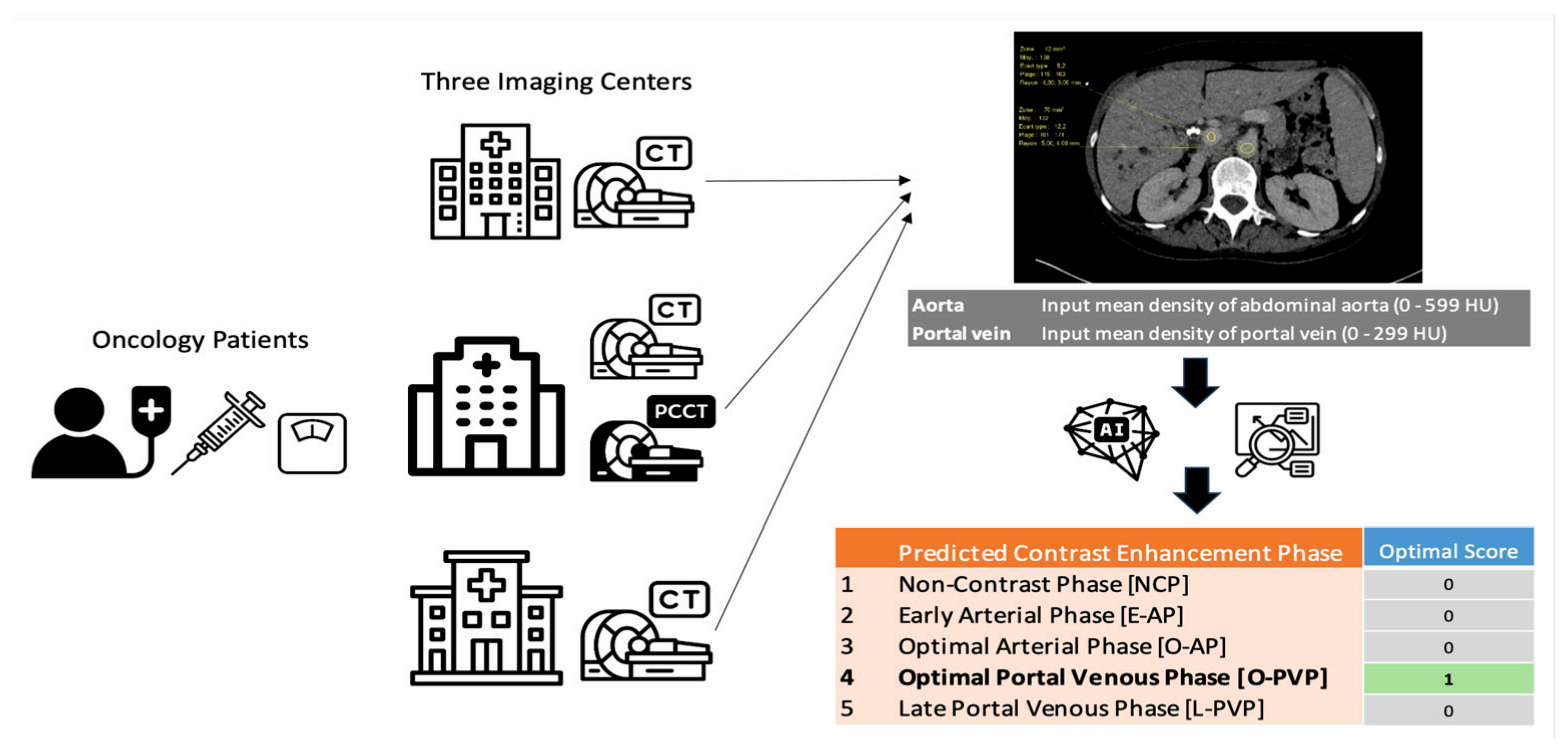
Overview of the study workflow for automated assessment of contrast-enhanced CT quality using the CECT-QC algorithm. Schematic representation of the study process. Patients enrolled in phase I oncology trials underwent contrast-enhanced CT examinations across three different imaging centers. CT data of aortic and portal vein attenuation values were processed by the CECT-QC algorithm, which automatically classifies each scan into one of five enhancement phases (non-contrast, early arterial, optimal arterial, optimal portal venous, or late portal venous). Scans were classified as optimal (phase 4) or non-optimal (other phases).

A previously validated AI CECT-QC algorithm [3,21] was employed to autonomously identify standardized vascular regions of interest in the aorta and portal vein, quantify contrast enhancement in Hounsfield Units (HU), and classify each examination into one of five contrast-enhancement phases using a random-forest classifier. The algorithm operates on routine DICOM CT images and automatically identifies the vascular regions of interest (ROIs), as illustrated in Figure 1. These quantitative measures served as proxies for vascular parenchymal enhancement. All CT examinations were independently reviewed by two radiologists, one senior and one junior. The radiologists assessed contrast injection adequacy, artifacts, visualization of relevant anatomical structures, appropriateness of the diagnostic phase, visual contrast, signal-to-noise ratio (SNR), contrast-to-noise ratio (CNR), and overall suitability for the clinical question. Predefined ROI measurements were performed before reviewing the CECT-QC output and independently of the algorithmic classification. The radiologists subsequently reviewed the CECT-QC phase classification. Three discrepancies were identified between the radiological assessment and the CECT-QC classification; in these cases, the assessment of the senior radiologist was retained as the final reference classification. Thus, the final reference classification was not based exclusively on the unmodified CECT-QC output. The algorithm assigned a phase-based classification using a standardized 5-point scale: (1) Non-Contrast Phase, (2) Early Arterial Phase, (3) Optimal Arterial Phase, (4) Optimal PVP, and (5) Late PVP [3,21]. The CECT-QC algorithm uses a random forest classifier comprising 10 trees with a maximum depth of 5, trained using aortic and portal venous attenuation values. The reference standard used for training was derived from continuous time-after-contrast curves and discretized into the five contrast-enhancement phases described above. In an independent test set, the classifier achieved an overall accuracy of 79.4% (95% CI, 75.2–82.9%), with phase-specific accuracies of 98% for non-contrast, 90% for optimal arterial, and 84% for optimal PVP classification [21]. For the present study, the five-phase scale was converted into a binary outcome, with phase 4 considered optimal PVP and all other phases considered non-optimal. The overall workflow is illustrated in Figure 2. Quantitative aortic and portal venous attenuation values were also recorded.

### 2.4. Statistical Analysis

The primary endpoint was the proportion of standard CT examinations with a final reference classification of optimal PVP enhancement, excluding PCCT examinations from the primary analysis. CECT-QC provided the initial algorithm-derived phase classification, which underwent radiological review as described above. Secondary endpoints included a series of multivariable logistic regression models adjusted for age, sex, and BMI. All models were restricted to standard CT examinations, with the exception of the scanner type comparison in Institution A, the only center with both scanner types. These models assessed independent associations between PVP optimality and: (i) scanner type (standard CT vs. PCCT, in Institution A), (ii) legislative period (pre- vs. post-1 March 2024, across French Institutions A and B, including an interaction term between legislative period and institution), and (iii) institution (comparing all three institutions, with Institution A as the reference). Results are reported as adjusted odds ratios (aORs) with 95% confidence intervals (CIs), and overall categorical effects were evaluated using likelihood ratio tests. Univariate analyses evaluated patient characteristics (age, sex, BMI) and contrast enhancement parameters (ICA type and weight-adjusted iodine load). In addition, iodine load per body weight was compared across five subgroups (Institution A pre- and post-legislation, Institution B pre- and post-legislation, and Institution C) using one-way ANOVA. Quantitative aortic and portal venous attenuation values were also compared between scanner types using unpaired *t*-tests.

Categorical variables are presented as counts and percentages, and continuous variables as mean ± standard deviation (SD). Differences in proportions were evaluated using chi-square or Fisher’s exact tests, as appropriate. Continuous variables were compared between two groups using unpaired Student’s *t*-tests or Wilcoxon rank-sum tests, and across more than two groups using one-way analysis of variance (ANOVA) after confirmation of normal distribution assumptions. All tests were two-tailed, and a *p*-value < 0.05 was considered statistically significant. Statistical analyses were performed using SciPy (version 1) in Python (version 3.12).

## 3. Results

### 3.1. AI Classification and Imaging Inputs

**Figure 3:**
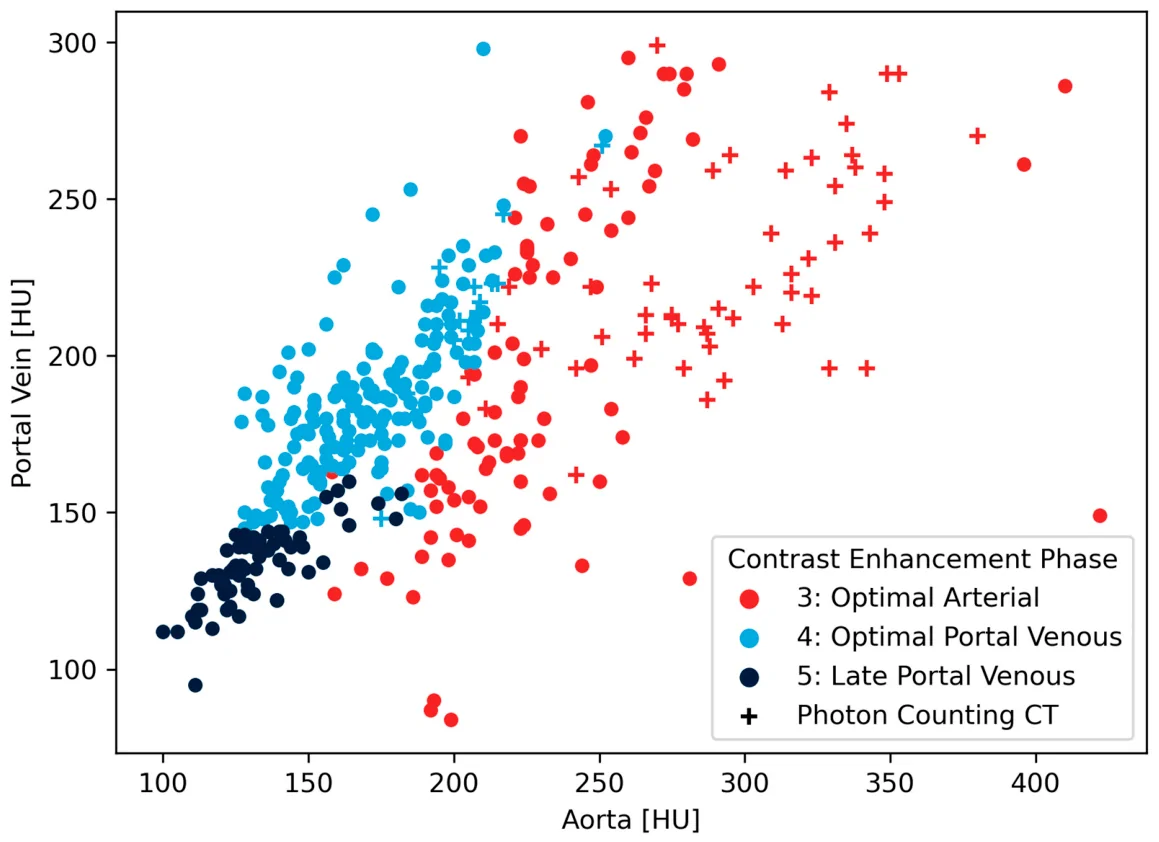
Contrast Enhancement Phase Determined using AI Algorithm. Scatter plot of aortic versus portal venous attenuation values [HU] across CT scans. Points are color-coded according to enhancement phase classification (optimal arterial, optimal portal venous, late portal venous). Cross-marked points (+) indicate scans performed with photon-counting CT (PCCT).

**Figure 4:**
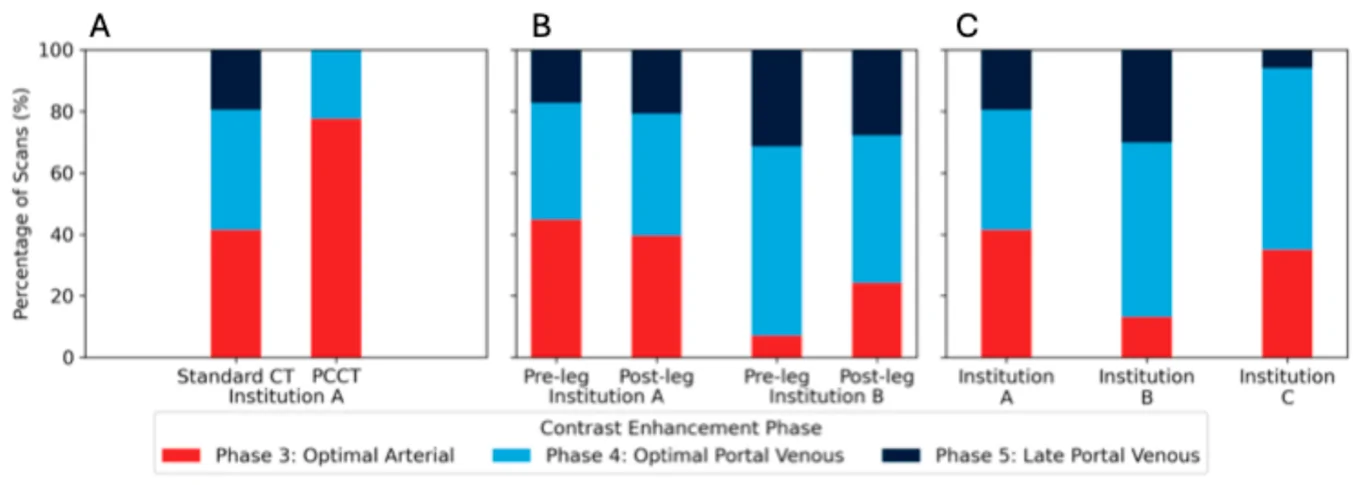
AI-Classified Contrast Enhancement Phase Distribution Stratified by Scanner Type, Legislative Period, and Institution. Stacked bar plots showing the distribution of AI-classified contrast enhancement phases (optimal arterial, optimal portal venous, and late portal venous) by CT scanner type at Institution A (Panel (**A**)), French legislative period at Institutions A and B (Panel (**B**)), and institution (Panel (**C**)). Panels (**B**,**C**) include standard CT examinations only, excluding PCCT examinations.

The AI algorithm assigned contrast enhancement phase scores to all 402 scans based on two quantitative inputs: aortic and portal venous attenuation (HU). Figure 3 displays the scatter plot of all scans in this two-dimensional input space, with each point colored by assigned phase, illustrating the decision boundaries between enhancement categories. PCCT scans, denoted by cross markers, clustered at higher aortic and portal venous attenuation values and were predominantly classified as optimal arterial phase (phase 3). Figure 4 shows the full distribution of phase assignments, spanning phases 3 (Optimal Arterial Phase), 4 (Optimal PVP), and 5 (Late PVP) stratified by CT scanner type, legislative period, and institution.

### 3.2. Overall Rates of Optimal CT Scan

Among standard CT examinations, 53.1% (178/335; 95% CI 47.8–58.4) were classified as optimal PVP (Table 1).

### 3.3. Association with Healthcare Setting

Among standard CT examinations, optimal PVP rates were 39.0% (32/82) at Institution A, 56.9% (87/153) at Institution B, and 59.0% (59/100) at Institution C. Institution was associated with optimal PVP classification in the multivariable analysis (overall likelihood-ratio test, χ^2^ = 6.48, *p* = 0.039). Compared with Institution A, the adjusted odds of optimal PVP classification were higher at Institution B (OR 1.88, 95% CI 1.07–3.29; *p* = 0.027; Table 1) and Institution C (OR 2.06, 95% CI 1.12–3.79; *p* = 0.020), after adjustment for age, sex, and BMI (Table 1).

### 3.4. Association with French Legislation

Among standard CT examinations from Institutions A and B, the multivariable temporal analysis showed no statistically significant association between time period (pre- versus post-legislation) and optimal PVP classification (OR 1.17, 95% CI 0.50–2.77; *p* = 0.716; Table 1). The post-legislation × institution interaction was also not statistically significant (OR 0.68, 95% CI 0.23–2.02; *p* = 0.486; Table 1), indicating no evidence that the change over time differed significantly between Institutions A and B after adjustment for age, sex, and BMI.

### 3.5. Association with CT Scanner Technology

**Figure 5:**
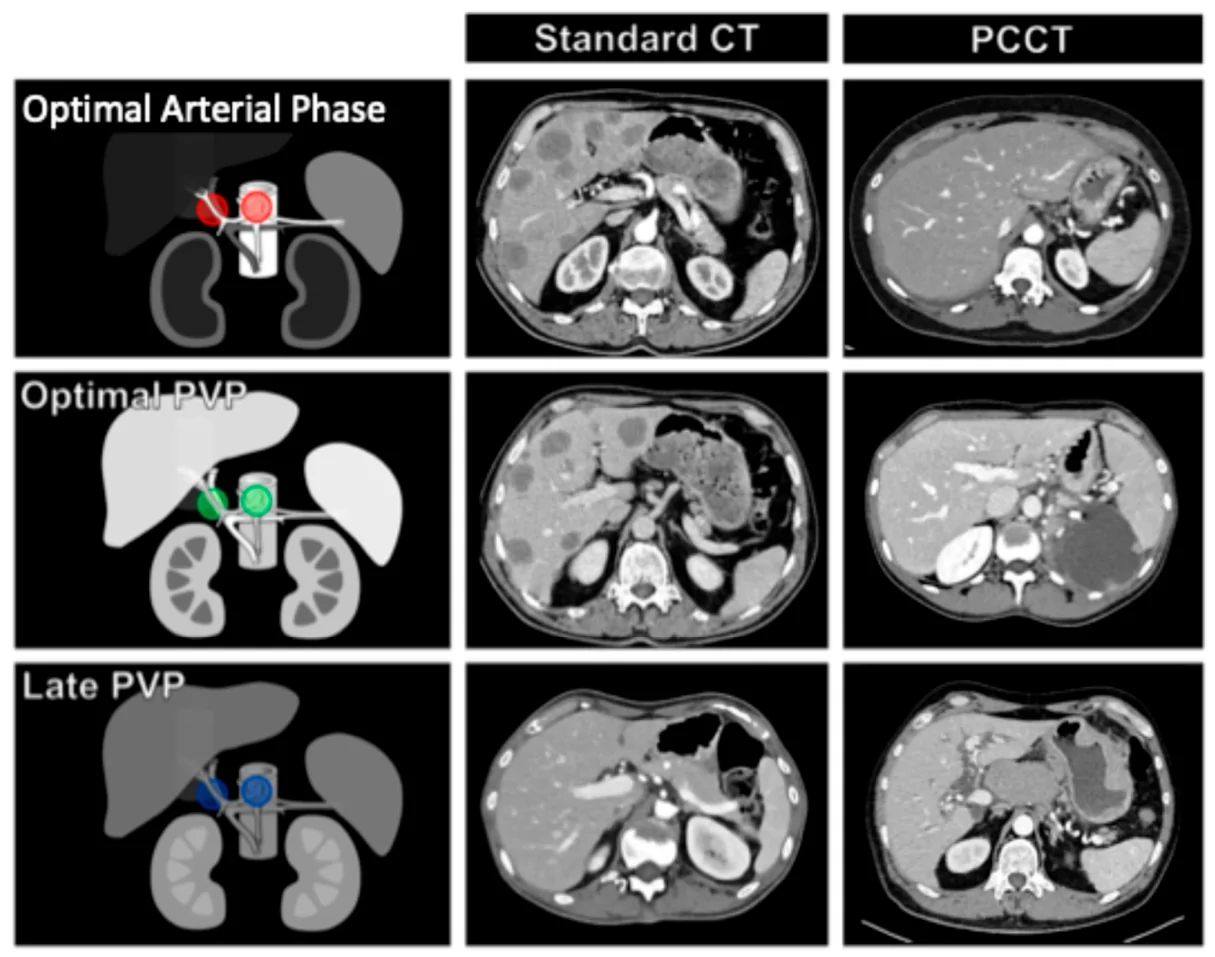
Standard CT versus PCCT AI algorithm outputs. Representative axial abdominal CT images are shown side-by-side for three contrast enhancement phases: Phase 3—Optimal Arterial Phase (**top row**), Phase 4—Optimal PVP (**middle row**), and Phase 5—Late PVP (**bottom row**). Schematics (**left column**) illustrate the corresponding expected vascular enhancement pattern for each phase.

Within Institution A, standard CT was associated with higher odds of optimal PVP classification compared with PCCT after adjustment for age, sex, and BMI (OR 2.24, 95% CI 1.07–4.68 for standard CT vs. PCCT; *p* = 0.031; Table 1). This difference is consistent with the higher vascular attenuation values observed on PCCT: mean aortic attenuation was 273.7 ± 52.3 HU on PCCT versus 182.6 ± 43.4 HU on standard CT (*p* < 0.001), and mean portal venous attenuation was 225.3 ± 30.8 HU versus 165.7 ± 30.9 HU, respectively (*p* < 0.001), resulting in predominant classification as optimal arterial phase (phase 3) rather than optimal PVP. Figure 5 shows side-by-side comparisons between standard CT and PCCT spanning contrast enhancement phases 3 (Optimal Arterial Phase), 4 (Optimal PVP), and 5 (Late PVP). PCCT demonstrates higher attenuation overall.

### 3.6. Association with Patient Characteristics

Optimal and non-optimal groups differed in mean age (67.2 ± 12.2 vs. 63.6 ± 12.8 years; *p* = 0.004) and BMI distribution (*p* = 0.035), with obese patients (BMI > 30) overrepresented in the non-optimal group. Sex was comparable between groups (*p* = 0.267; Table 2).

In the multivariable analyses, age and BMI were associated with optimal PVP classification. In the temporal comparison, older age was associated with higher odds of optimal PVP classification (OR 1.04 per year, 95% CI 1.01–1.06; *p* = 0.005), while higher BMI was associated with lower odds of optimal PVP classification (OR 0.93 per unit, 95% CI 0.88–0.99; *p* = 0.022). In the institution comparison, BMI remained associated with optimal PVP classification (OR 0.95 per unit, 95% CI 0.89–1.00; *p* = 0.047), whereas age was not statistically significant (OR 1.01, 95% CI 0.99–1.03; *p* = 0.250). Sex was not significantly associated with optimal PVP classification in either model (Table 1).

### 3.7. Association with Iodine Contrast

**Figure 6:**
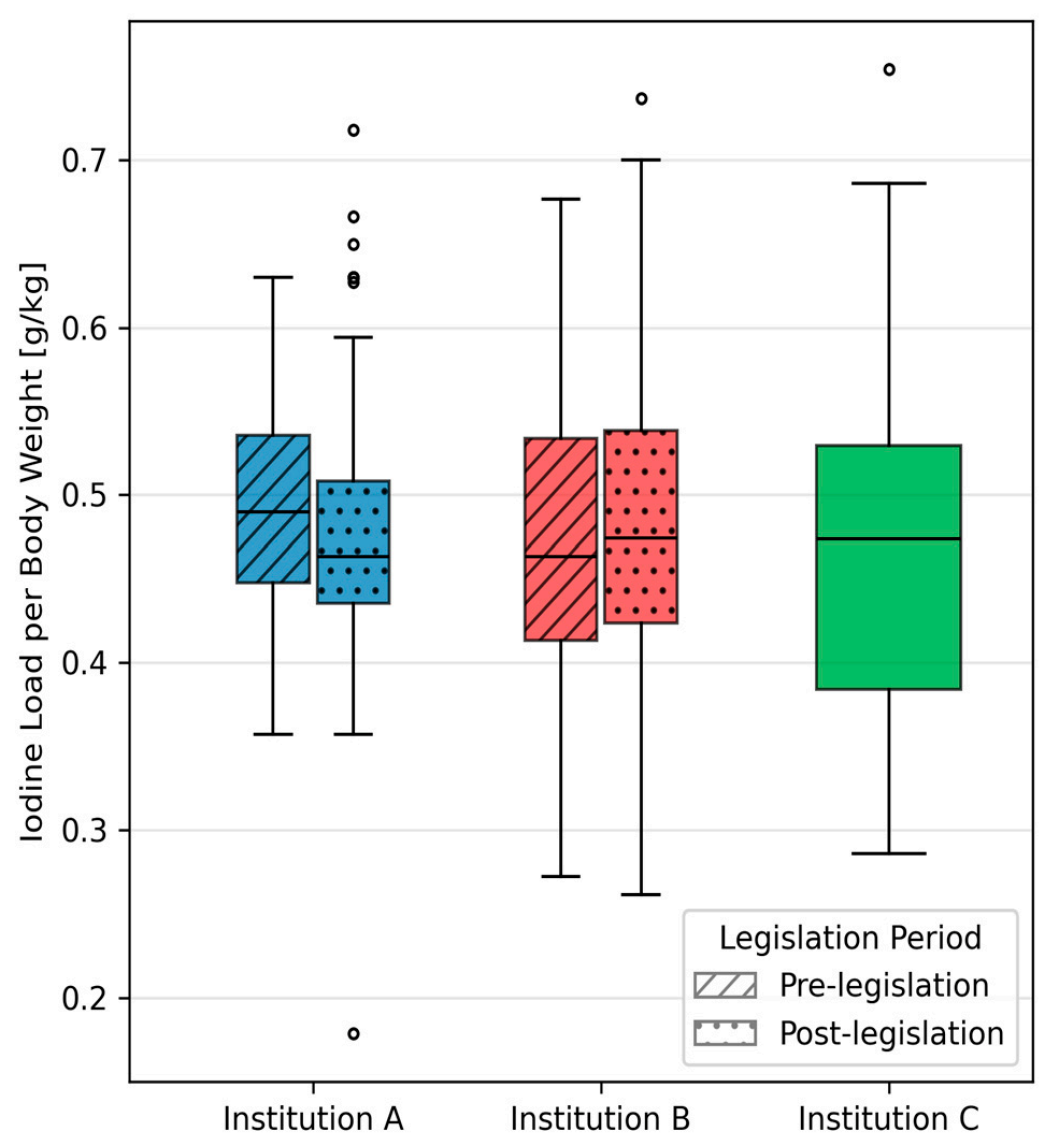
Iodine Load per Body Weight by Institution and Legislative Period. Box plot of iodine contrast dose load per body weight [g/kg] stratified by institution and legislative period. No statistically significant difference was observed across institutions or pre- vs. post- legislation.

Iodine load per body weight was similar across the five different institution-legislative subgroups (*p* = 0.45), as illustrated in Figure 6, and did not differ between optimal and non-optimal scans (*p* = 0.72; Table 2). No significant association was found between ICA type and scan optimality (*p* = 0.22; Table 2).

## 4. Discussion

In this multicenter study evaluating PVP contrast-enhanced CT examinations in patients enrolled in phase I oncology trials, 53.1% of standard CT examinations were classified as optimal PVP. Imaging quality and phase consistency remain important for response assessment under RECIST 1.1 and for quantitative imaging endpoints [10,22]. In early-phase drug development, imaging is often a primary surrogate marker of efficacy and safety [9,11,12], emphasizing the value of consistent CT acquisition across participating institutions [11,23]. Although there are inter-institutional differences in optimal scan rates and organ contrast enhancement densities, all institutions and clinical trials could benefit from quality improvement initiatives.

A secondary focus of this study was to explore temporal changes in PVP classification before and after the implementation of France’s Social Security Financing Act (LFSS 2024), which authorized the use of multi-patient contrast injectors and transferred the burden of cost of ICAs from patients to radiologists and radiology centers. Our results showed no statistically significant changes in optimal scan rates before and after legislation at institution A or B. A non-significant decline in optimal scan rates was observed at the private French radiology department (Institution B). However, detailed information on potential changes in contrast injection protocols, including contrast volume, injection rate, and timing, was not systematically available. Therefore, this pre/post comparison should be considered exploratory and no causal relationship between the legislation and the observed temporal changes can be established. Ongoing surveillance is warranted to evaluate whether such temporal trends are associated with changes in contrast delivery practices.

Patient characteristics also showed associations with optimal PVP classification.

In univariate analysis, younger age and higher BMI were associated with non-optimal scans, with obese patients (BMI > 30) overrepresented in the non-optimal group. In adjusted analyses, older age was associated with higher odds of optimal PVP classification in the temporal model, while higher BMI was associated with lower odds of optimal classification in both the temporal and institutional models. This is physiologically plausible, as increased body habitus may alter contrast distribution and hepatic perfusion dynamics, affecting the timing of portal venous enhancement [7].

Iodine load per body weight was similar across institutional and legislative subgroups and was not associated with optimal PVP classification. Injection rate and scan timing may contribute to PVP enhancement; however, these variables were not systematically available in the present retrospective cohort, and their respective effects could not be assessed. Moreover, the absence of a statistically significant association between iodine load and optimal PVP classification should not be interpreted as evidence that PVP classification is independent of iodine quantity. Prospective studies systematically recording injection parameters and scan timing are needed to further investigate these relationships. Prior work in metastatic colorectal cancer showed that non-optimal PVP timing was associated with an approximately 15% decrease in measured tumor attenuation compared with optimal PVP, illustrating the potential clinical relevance of consistent contrast-phase acquisition [3]. This outcome was not assessed in the present study.

**Figure 7:**
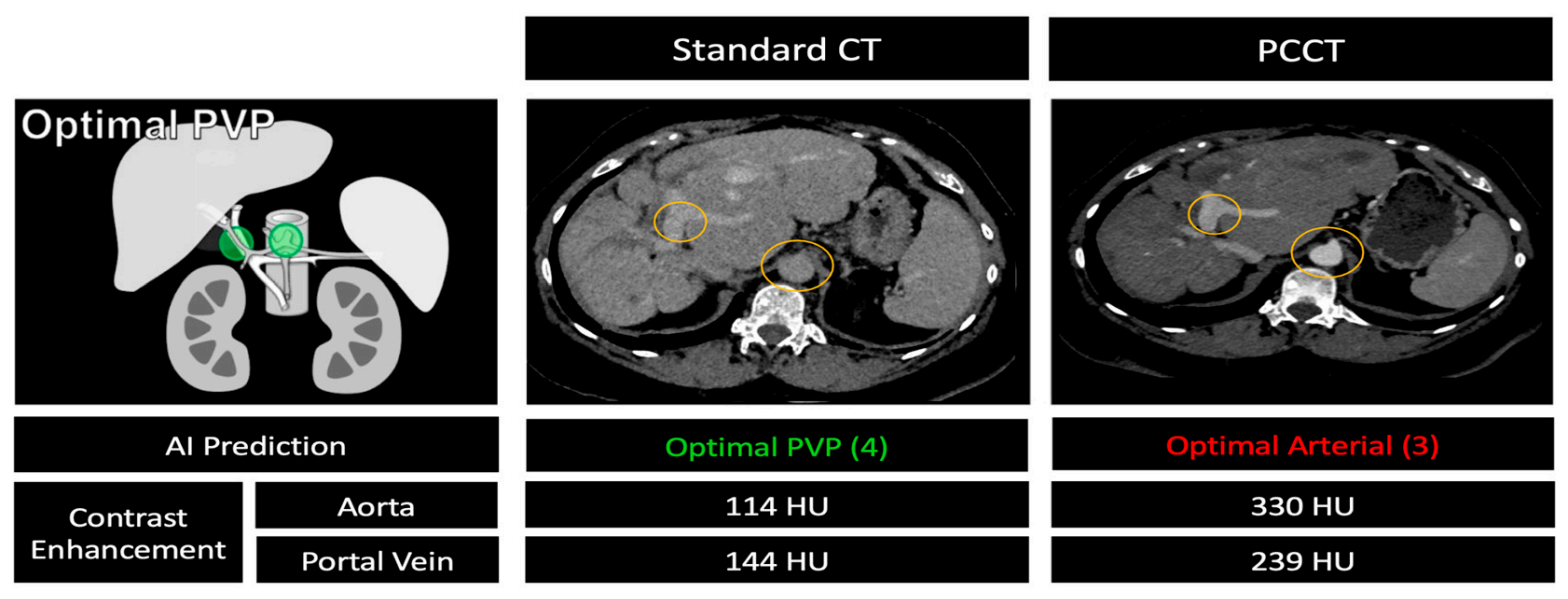
Example of standard CT and PCCT examinations acquired during optimal PVP with different CECT-QC classification outputs. On radiological assessment, both examinations were considered to represent optimal PVP. The standard CT examination was classified by CECT-QC as optimal PVP (phase 4), whereas the PCCT examination was classified as optimal arterial phase (phase 3), in the setting of markedly higher vascular attenuation values. Yellow circles indicate approximate vascular regions of interest.

Recent advancements in PCCT have markedly improved CT image quality and diagnostic performance [24]. At Institution A, however, PCCT examinations were frequently classified by CECT-QC as optimal arterial phase (score 3) rather than optimal PVP (score 4), as demonstrated in Figure 7. This may partly reflect the fact that the AI algorithm was not trained on PCCT data. In our cohort, PCCT examinations showed higher aortic and portal venous attenuation than conventional CT, as exemplified in Figure 5. However, this finding should not be attributed to scanner technology alone, as reconstruction method, acquisition energy, scanner platform, and contrast timing may all have contributed to the observed differences. All PCCT examinations were acquired using a standardized oncologic thoraco-abdomino-pelvic protocol and consistently reconstructed as VMI at 70 keV. Importantly, algorithmic misclassification does not indicate reduced image quality, but rather a limitation of applying a classification tool developed for standard CT to newer imaging technology. While Institution A currently administers the same standardized contrast volumes across scanner types, Layer et al. reported contrast-dose reduction with PCCT while preserving image quality and diagnostic interpretability [25]. Furthermore, van der Bie et al. described improved spatial resolution, energy-resolved imaging, and reduced radiation exposure with PCCT [26]. These findings highlight the need for ongoing retraining and validation of quality-control tools as imaging technologies evolve.

Malafosse et al. evaluated the medico-economic and environmental impact of the 1 March 2024 decree [19] introducing multi-patient injectors in CT suites. In a large university hospital, switching from single- to multi-patient systems reduced unused iodinated contrast by 77% (96 L to 17 L), plastic waste by 55% (~5.4 tons/year), and generated annual savings of €35,000 across three scanners, along with an estimated 30-ton reduction in CO_2_ emissions [20]. These findings suggest that multi-patient injection systems may reduce both resource use and costs, although their broader environmental and clinical impact requires multicenter evaluation.

This study has several limitations. First, the AI algorithm used to classify enhancement phases was not trained on PCCT data, limiting its applicability to these next generation scanners. Second, the non-French tertiary center had a limited number of post-legislation scans, precluding its use as a time-based control to assess effects of French legislation. Third, injection rate and scan timing were not systematically available across institutions, and individual tube-voltage data were unavailable, precluding adjustment for these factors and assessment of tube-voltage distributions, which may influence iodine attenuation and CECT-QC classification. Fourth, the pre- and post-legislation analysis should be interpreted as an exploratory assessment of temporal changes rather than evidence of a causal effect of the legislation, particularly given the absence of a contemporaneous control group and direct measurement of protocol changes in a retrospective observational study. Finally, although multivariable analyses adjusted for age, sex, and BMI, tumor distribution was not included in the models because several tumor categories had small numbers within individual institutions, resulting in sparse strata and potentially unstable estimates.

## 5. Conclusions

Among standard CT examinations in phase I oncology trials, 53.1% were classified as optimal PVP contrast enhancement, with significant performance variability across institutions. Our findings highlight the potential role of scalable quality control tools, such as AI-based classifiers and institutional performance monitoring across sites. Future studies should evaluate whether protocol harmonization and appropriate quality control tools can increase the proportion of examinations acquired during an optimal PVP.

## Figures and Tables

**Table 1:** Multivariable logistic regression analyses of factors associated with optimal portal venous phase (PVP) timing. Odds ratios (ORs) and 95% confidence intervals (CIs) were estimated using logistic regression models adjusted for age, sex, and body mass index (BMI). Scanner type comparison was restricted to Institution A, the only institution equipped with photon-counting CT (PCCT). Temporal comparison was restricted to standard CT examinations. The institution comparison was also restricted to standard CT examinations, with Institution A as the reference category. *p* < 0.05 was considered statistically significant.

Analysis	Variable	N Total	Optimal, n (%)	OR (95% CI)	*p*-Value
Overall optimal rate (standard CT only)	Overall optimal rate	335	178 (53.1%)	53.1% (47.8–58.4%)	
Scanner type comparison (Institution A only)	Standard CT vs. PCCT	149	47 (31.5%)	2.24 (1.07–4.68)	0.031 *
	Age (years)			1.02 (0.98–1.06)	0.330
	Male vs. Female			0.89 (0.44–1.82)	0.756
	BMI (kg/m^2^)			0.97 (0.90–1.05)	0.449
Temporal comparison (standard CT only)	Post- vs. pre-legislation	302	134 (44.4%)	1.17 (0.50–2.77)	0.716
	Institution B vs. Institution A			3.03 (1.23–7.47)	0.016 *
	Post-legislation × institution			0.68 (0.23–2.02)	0.486
	Age (years)			1.04 (1.01–1.06)	0.005 *
	Male vs. Female			0.86 (0.52–1.40)	0.541
	BMI (kg/m^2^)			0.93 (0.88–0.99)	0.022 *
Institution comparison (ref = Institution A; standard CT only)	Institution B vs. Institution A	335	178 (53.1%)	1.88 (1.07–3.29)	0.027 *
	Institution C vs. Institution A			2.06 (1.12–3.79)	0.020 *
	Age (years)			1.01 (0.99–1.03)	0.250
	Male vs. Female			1.02 (0.65–1.61)	0.927
	BMI (kg/m^2^)			0.95 (0.89–1.00)	0.047 *
	Overall institution effect (LR test, 2 df)			χ^2^ = 6.48	0.039 *

* *p* < 0.05 indicates statistical significance.

**Table 2:** Patient and contrast protocol characteristics associated with optimal portal venous phase (PVP) timing. Data are presented as n (%) or mean ± standard deviation. Optimal PVP was defined according to the final reference phase classification after radiological verification of the CECT-QC output. *p*-values were calculated using chi-square or Fisher’s exact tests for categorical variables and Student’s *t*-test or Wilcoxon rank-sum test for continuous variables, as appropriate (* significant at *p* < 0.05).

Characteristic	Optimal PVP	Non-Optimal PVP	*p*-Value
**Patient characteristics**			
Age (years)	67.2 ± 12.2	63.6 ± 12.8	0.004 *
Sex—Female	78 (40.4%)	97 (46.4%)	0.267
Sex—Male	115 (59.6%)	112 (53.6%)	
BMI < 19	10 (5.2%)	7 (3.3%)	0.035 *
BMI 19–25	110 (57.0%)	110 (52.6%)	
BMI 25–30	60 (31.1%)	59 (28.2%)	
BMI > 30	13 (6.7%)	33 (15.8%)	
**Contrast enhancement protocol**			
ICA type—Xenetix	75 (38.9%)	69 (33.0%)	0.22
ICA type—Omnipaque	67 (34.7%)	90 (43.1%)	
ICA type—Iomeron	31 (16.1%)	39 (18.7%)	
ICA type—Other	20 (10.4%)	11 (5.3%)	
ICA load per body weight (g/kg)	0.471 ± 0.090	0.475 ± 0.088	0.72

## Data Availability

Data supporting the findings of this study are available from the corresponding author upon reasonable request.

## Appendix A

**Table A1:** Demographic Data, Scanner Types and Tumor Characteristics of Patients Across the Three Institutions. Summary of demographics, scanner types, and primary tumor sites for patients undergoing CT imaging at Institution A, Institution B, and Institution C. Data are shown as n (%).

Variable	Institution A	Institution B	Institution C
Number of scans (n)	149	153	100
Age (mean ± SD)	61.7 ± 9.3	67.2 ± 12.1	67.9 ± 16.1
Sex—Female	75 (50.3%)	59 (38.6%)	41 (41%)
Sex—Male	74 (49.7%)	94 (61.4%)	59 (59%)
CT scanner—Standard	82 (55%)	153 (100%)	100 (100%)
CT scanner—Photon-counting	67 (45%)	0	0
Tumor—Digestive	24 (16.1%)	30 (19.6%)	61 (61%)
Tumor—Gynecologic	19 (12.8%)	9 (5.9%)	9 (9%)
Tumor—Lung	42 (28.2%)	78 (51%)	5 (5%)
Tumor—Breast	3 (2%)	21 (13.7%)	3 (3%)
Tumor—Urologic	16 (10.7%)	5 (3.3%)	17 (17%)
Tumor—Lymphoma	23 (15.4%)	3 (2%)	4 (4%)
Tumor—Kidney	10 (6.7%)	1 (0.7%)	0
Tumor—Head and neck	6 (4%)	3 (2%)	0
Tumor—Melanoma	4 (2.7%)	0	0
Tumor—Other	2 (1.3%)	3 (2%)	1 (1%)
